# Supplementary material for: Delivery determinants of an Acinetobacter baumannii type VI secretion system bifunctional peptidoglycan hydrolase
Source: mBio. 2024 Dec 31;16(2):e02627-24. doi: 10.1128/mbio.02627-24 (PMC11796386; doi:10.1128/mbio.02627-24)
Supplement: Supplemental material — Supplemental figures, table, and movie legend. [file mbio.02627-24-s0001.docx]

**Figure S1**


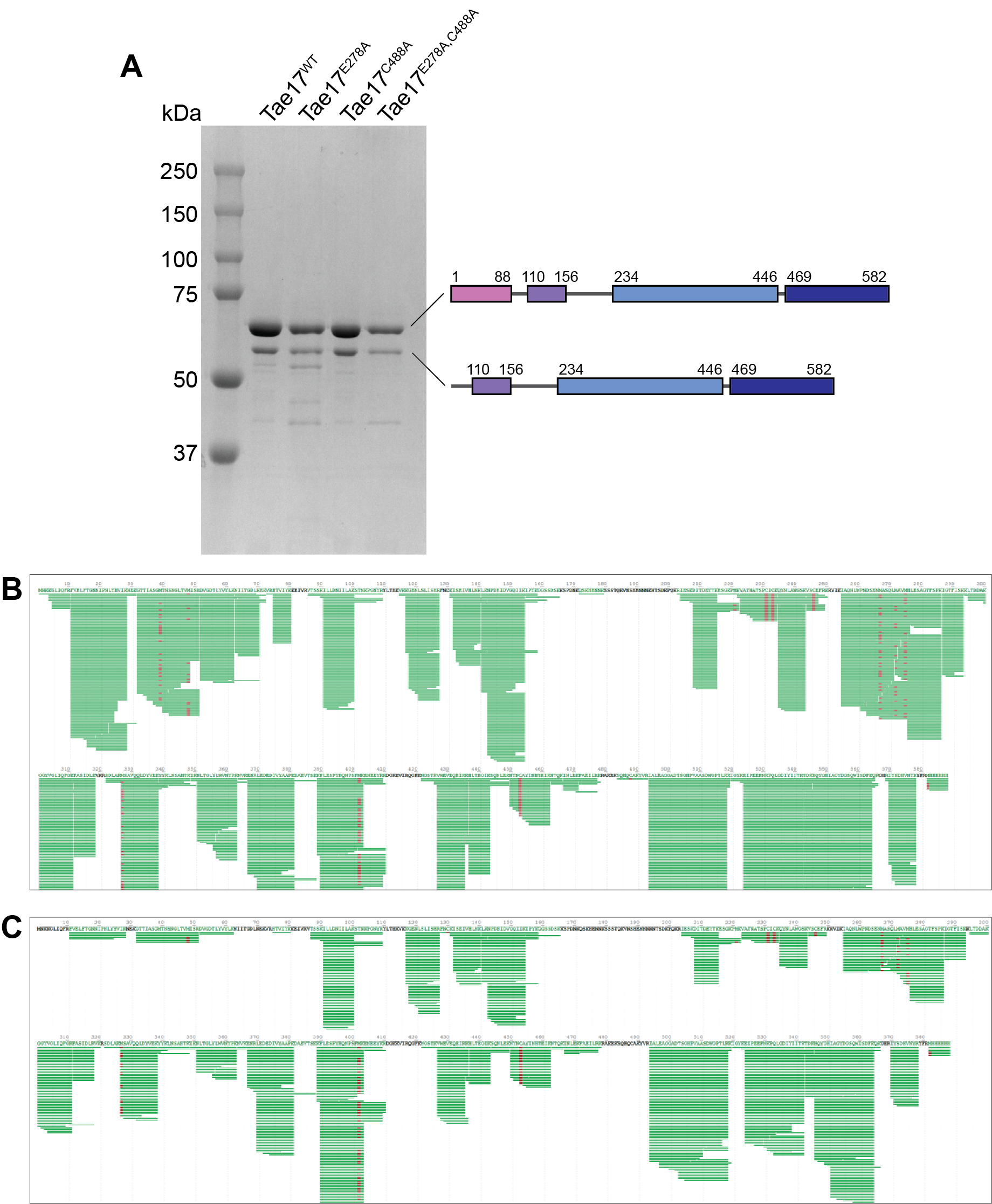


**Figure S1** **Purification of Tae17 and active site mutants.** (**A**) All proteins were purified by metal-affinity chromatography followed by size-exclusion chromatography. PAGE of Tae17^WT^, Tae17^E278A^, Tae17^C488A^ and Tae17 ^E278AC488A^. The proposed domain structure of the two major species is shown at the right. Size markers are shown at the left. (**B**) Mass spectrometry peptide coverage of full-length Tae17. (**C**) Mass spectrometry peptide coverage of N-terminally truncated Tae17. For both (B) and (C) green bars represent coverage across those residues, multiple bars indicate replicate coverage.

# **Figure S2**

**
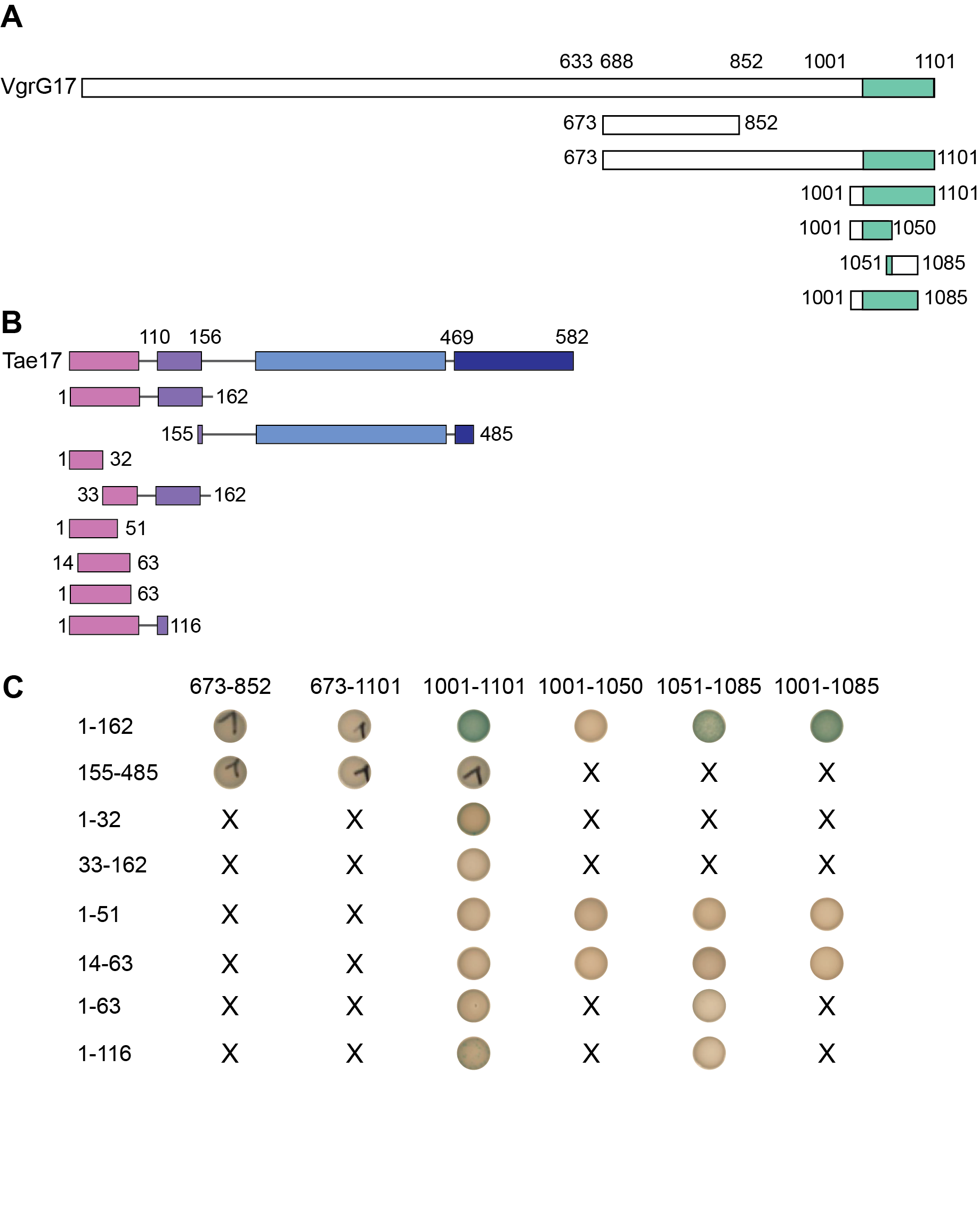
**

**Figure S2 Bacterial adenylate cyclase two-hybrid analysis of the interacting regions of VgrG17 and Tae17.** (**A**) Schematic representation of VgrG17 and the various regions used in the bacterial two-hybrid interaction studies. The white region indicates the conserved region of VgrG predicted to participate in the formation of the stalk formed by the trimerization of VgrG proteins at the T6SS tip. The C-terminal end of the protein (green) is unique to VgrG17 and specific regions within it are predicted to interact with Tae17 for its delivery. (**B**) A schematic representation of Tae17 and the regions that were assessed for their direct interaction with VgrG17 using bacterial adenylate cyclase two hybrid experiments. Domains are coloured according to structure in Figure 1. (**C**) Figure showing T18-VgrG17 and T25-Tae17 proteins tested for their interaction. Numbers on the left indicate region of Tae17 fused to T25 adenylate cyclase fragment. Numbers at top of figure indicate region of VgrG17 fused to the T18 fragment of adenylate cyclase. The presence of interaction between pairs of proteins in the assay is indicated by the formation of blue colonies on selective media (actual colony images are shown). Text crosses indicate VgrG17 and Tae17 region pairs that were not tested.

**Figure S3**

**
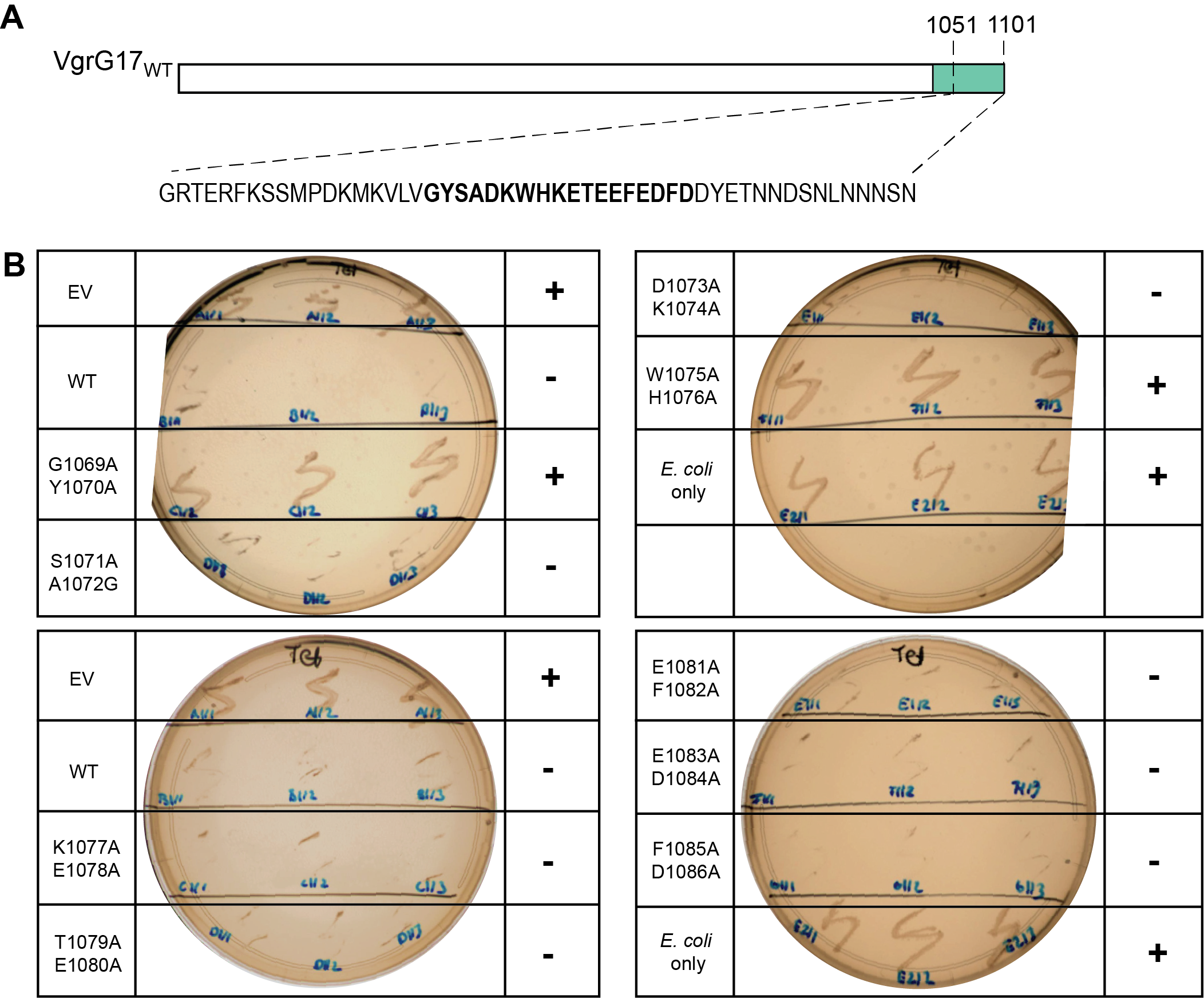
**

**Figure S3. Alanine scanning mutagenesis of VgrG17 to determine the amino acids important for delivery of effector Tae17.** (A) Schematic representation of VgrG17 with amino acids 1051-1101 shown. The amino acids shown in bold were included in the pair-wise alanine mutagenesis in this study. Numbers indicate amino acid positions. (B) A qualitative interbacterial killing assay with AB307-0294 Δ*tse15*Δ*tde16*Δ*vgrG17* as predator and *E. coli* DH10B harbouring empty pWH1266 vector (for selection) as prey. *E. coli* prey was cultured alone on solid media or co-cultured with *A. baumannii* predator containing empty vector (EV), or the plasmids expressing wild-type VgrG17 (WT), or VgrG17 proteins with double amino substitutions as shown. For clarity, presence (+) or absence (-) of growth is also indicated to the right of the plate image.

**Figure S4**

^
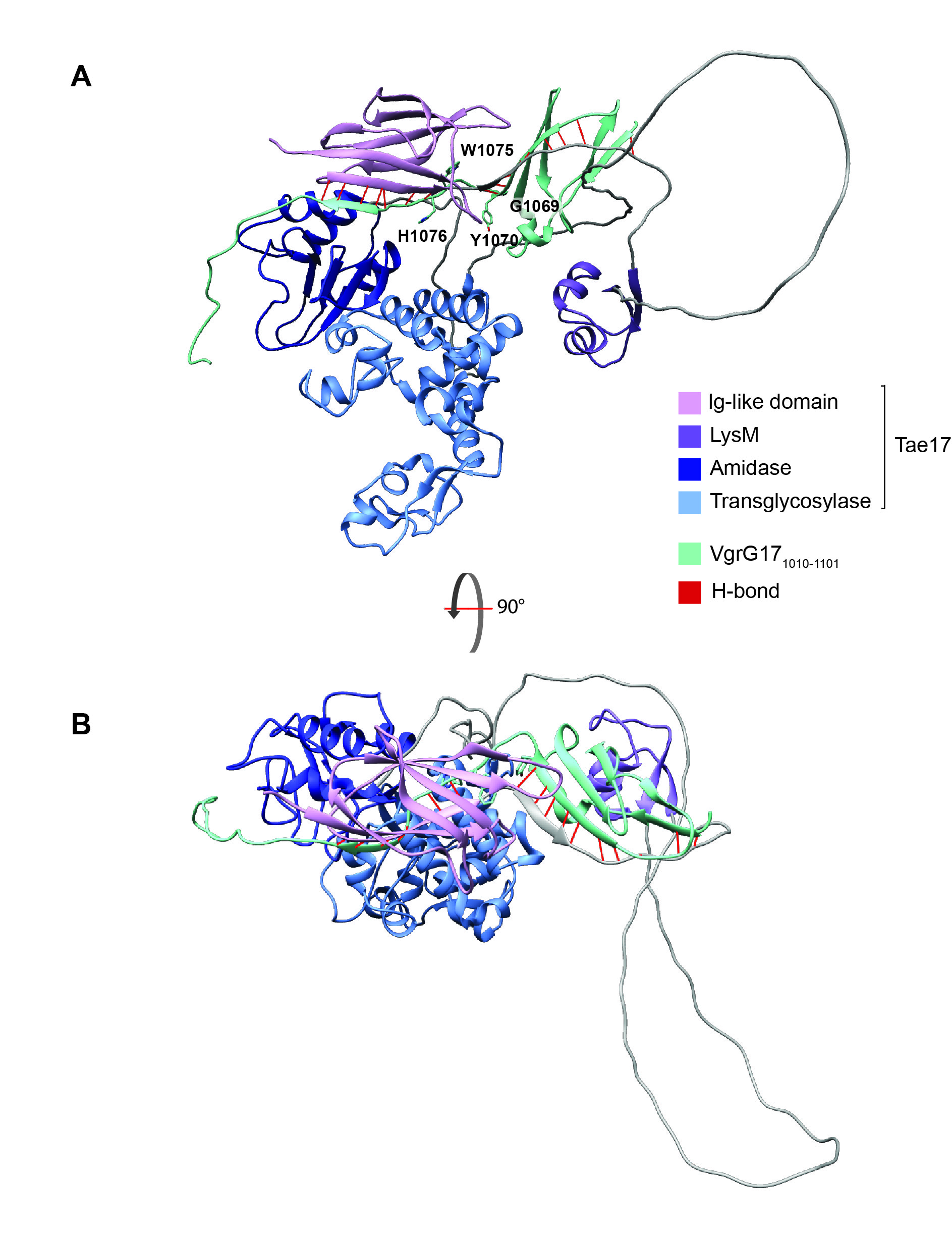
^

**Figure S4. AlphaFold2 model of VgrG17_1010-1101_ interacting with Tae17 with predicted hydrogen bonds.** For both **A**) and **B)** VgrG17 is shown in green, and the Tae17 Ig-like domain is shown in pink, the LysM domain is shown in purple, the lytic transglycosylase domain is shown in light blue and the amidase domain in dark blue. The linker regions are coloured grey. Edge-to-edge contact hydrogen bonds are shown in red. Residues of interest mutated during the alanine scanning mutagenesis are shown. **(B)** The same VgrG17_1010-1101_ interaction with full length Tae17 but rotated forward 90 degrees.

# **Figure S5**

**
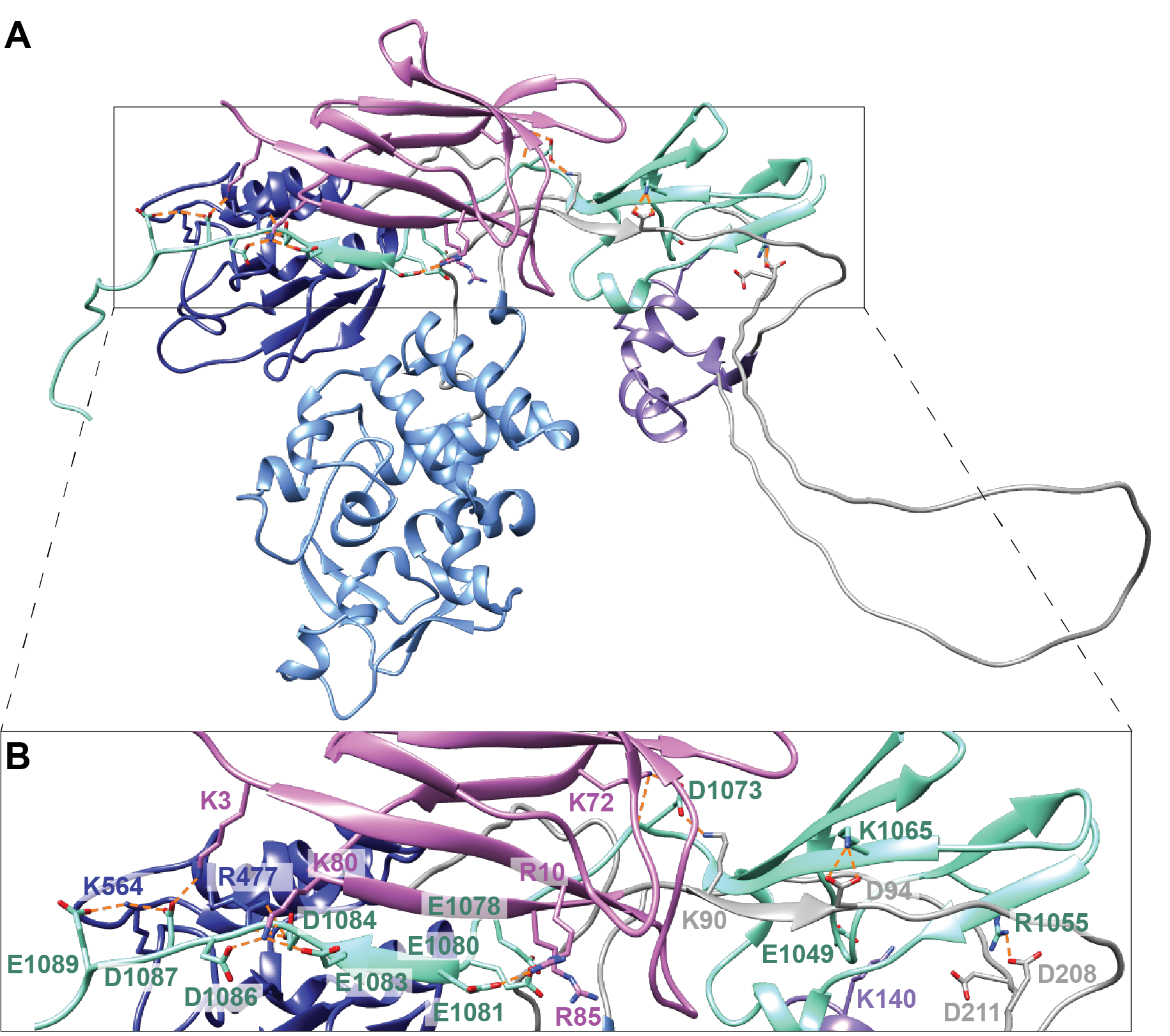
**

**Figure S5. AlphaFold2 model of VgrG17_1010-1101_ interacting with Tae17 with predicted salt bridges shown.** For both **A**) and **B**) VgrG17 is shown in green, Tae17 Ig-like domain is shown in pink, the LysM domain is shown in purple, lytic transglycosylase domain in light blue and amidase domain in dark blue. The linker regions are coloured grey. All residues predicted to form salt bridges by PISA are shown as sticks and further coloured by heteroatom. All salt bridges that can form between these residues, as determined by Chimera, are shown as dashed orange lines. All other PISA predicted salt bridges are possible following minor conformational changes, as such we have shown all residues even if bonds were not predicted using Chimera. **A**) shows the full length Tae17 and VgrG17_1010-1101_ with salt bridges. **B**) depicts a zoomed version of panel A) where each of the residues is also labelled using single letter code. The colour of the label identifies the chain or domain that the residue is found within. For further visualization of these interactions, please see the supplementary movie S1.

# **Table S1** VgrG17:Tae17 AlphaFold2 model salt bridges.

| **VgrG17 residue ^a^** | **Tae17 residue** |
| --- | --- |
| **Glu1049** | Lys140 |
| Arg1055 | **Asp208** |
|  | **Asp211** |
| Lys1065 | **Asp94** |
| **Asp1073** | Lys72 |
|  | Lys90 |
| **Glu1078** | Arg85 |
| **Glu1080** | Arg85 |
|  | Arg10 |
| **Glu1081** | Arg10 |
| **Glu1083** | Lys80 |
| **Asp1084** | Arg477 |
| **Asp1086** | Lys80 |
| **Asp1087** | Lys3 |
|  | Lys564 |
| **Glu1089** | Lys564 |

^a^ Bold font indicates negative charge, normal font indicates positive charge

Supplemental Movie S1 (separate file). Movie depicting a rotation of the AlphaFold2 model of VgrG17_1010-1101_ interacting with Tae17 with predicted salt bridges shown. VgrG17_1010-1101_ is shown in green. Tae17 is coloured by domain: the Ig-like domain is shown in pink, the LysM domain is shown in purple, the lytic transglycosylase domain in light blue, amidase domain in dark blue and linker regions are grey. All residues predicted to form salt bridges by PISA are shown as sticks and further coloured by heteroatom. All salt bridges that can form between these residues, as determined by Chimera, are shown as dashed orange lines. All other PISA predicted salt bridges are possible following minor conformational changes, as such we have shown all residues even if bonds were not predicted using Chimera.
